# Lessons from community participation in primary health care and water resource governance in South Africa: a narrative review

**DOI:** 10.1080/16549716.2021.2004730

**Published:** 2022-01-07

**Authors:** Jennifer Hove, Lucia D’Ambruoso, Kathleen Kahn, Sophie Witter, Maria van der Merwe, Denny Mabetha, Kingsley Tembo, Rhian Twine

**Affiliations:** a MRC/Wits Rural Public Health and Health Transitions Research Unit (Agincourt), School of Public Health, Faculty of Health Sciences, University of the Witwatersrand, Johannesburg, South Africa; b Aberdeen Centre for Health Data Science (Achds) Institute of Applied Health Sciences, School of Medicine, Medical Sciences and Nutrition, University of Aberdeen, Scotland, UK; c Department of Epidemiology and Global Health, Umeå University, Umeå, Sweden; d Public Health/Health Protection, National Health Service (NHS) Grampian, Scotland, UK; e International Network for the Demographic Evaluation of Populations and Their Health (Indepth), Accra, Ghana; f Institute for Global Health and Development, Queen Margaret University, Edinburgh, UK; g Independent Consultant, White river, South Africa; h Arthur Davison Children’s Hospital, Ndola, Zambia

**Keywords:** Community participation, primary health care, water governance, health committees, catchment management agencies

## Abstract

**Background:**

In South Africa, community participation has been embraced through the development of progressive policies to address past inequities. However, limited information is available to understand community involvement in priority setting, planning and decision-making in the development and implementation of public services.

**Objective:**

This narrative review aims to provide evidence on forms, extents, contexts and dynamics of community participation in primary health care (PHC) and water governance in South Africa and draw cross-cutting lessons. This paper focuses on health and water governance structures, such as health committees, Catchment Management Agencies (CMA), Water User Associations (WUAs), Irrigation Boards (IBs) and Community Management Forums (CMFs).

**Methods:**

Articles were sourced from Medline (Ovid), EMBASE, Google Scholar, Web of Science, WHO Global Health Library, Global Health and Science Citation Index between 1994 and 2020 reporting on community participation in health and water governance in South Africa. Databases were searched using key terms to identify relevant research articles and grey literature. Twenty-one articles were included and analysed thematically.

**Results:**

There is limited evidence on how health committees are functioning in all provinces in South Africa. Existing evidence shows that health committees are not functioning effectively due to lack of clarity on roles, autonomy, power, support, and capacity. There was slow progress in establishment of water governance structures, although these are autonomous and have mechanisms for democratic control, unlike health committees. Participation in CMAs/WUAs/IBs/CMFs is also not effective due to manipulation of spaces by elites, lack of capacity of previously disadvantaged individuals, inadequate incentives, and low commitment to the process by stakeholders.

**Conclusion:**

Power and authority in decision-making, resources and accountability are key for effective community participation of marginalized people. Practical guidance is urgently required on how mandated participatory governance structures can be sustained and linked to wider governance systems to improve service delivery.

## Background

Globally, community participation continues to be recognised as key in development, implementation and evaluation of programmes and interventions in health and other sectors. Dating back to 1978, the Alma-Ata Declaration on Primary Health Care (PHC) identified that ‘people have the right to participate individually and collectively in the planning and implementation of their health care’ [1]. In low- and middle-income countries (LMICs), community participation has been instrumental as a way of addressing barriers in health care service utilisation [2]. Community participation through collaborative action is thought to be critical in sustainability of programmes, importantly in low resource settings. Many LMICs have implemented community health worker (CHW) programs to address various health issues and to link marginalized communities with health systems [2–5].

In South Africa, community participation has been mooted as essential in addressing challenges of inequality, access, and poverty and in achieving health for all. Attempting to redress racial and class inequities, the Bill of Rights in section 27 of the Constitution of South Africa (1996) states that access to health care is a basic human right [6,7]. Since democracy in 1994, major changes in policy and legislation were made to improve access to quality health care and delivery of health services across the country, decentralise the district health systems, and promulgate participation in health service governance [8].

The National Health Act of 2003 sought to institutionalize community participation in PHC by advocating the establishment of health committees, hospital boards, and district health councils as health governance structures [8]. Provinces were given responsibility to establish health committees for PHC facilities and community health centres [8]. Seven of the nine provinces have legislation in place that permits the establishment of health committees by 2019 [7,8]. Implementation of health committees varies substantially across the nine provinces as provinces have the autonomy to apply different provincial policies or guidelines [9]. Even though post-apartheid development policies showed commitment to restructure public health services and embraced community participation, there are challenges in implementation [10].

Water is a social determinant of health, and is a major driver of health inequalities faced by people globally [11]. Communities are well aware of the link between access to safe water and health, as evidenced in earlier research in rural South Africa where community members nominated lack of safe water as their key health priority [12]. Access to safe water is also critical for health and well-being [13]. Despite mixed progress, South Africa has led the way in global water policy discourse: in 1996 the Right to Water was enacted as a constitutional right for all South Africans, 14 years before the United Nations 2010 declaration of water as a Basic Human Right [7]. This was followed by, *inter alia*, the 1997 National Water Policy White paper, Water Services Act (Act 108 of 1997) and National Water Act (Act 36 of 1998), considered among the most progressive water policies in the world [14–16].

As such, policies aimed at providing a robust national water resource management framework emphasised decentralization of water management, protection of water resources and stakeholder consultation and participation in governance [17,18]. Despite progressive health and water policies and a supportive legal framework, South Africa still suffers from poverty-related illness and severe shortages of safe water across the country [10,13]. In addition, considerable uncertainty remains on the forms, extents and contextual factors that influence the added value of community participation in development and implementation of policies and equitable provision of services.

Key to progressive policies in both PHC and water governance is the transformation of institutions and promotion of people-centred services through community participation. Implementation of community participation has proved difficult and is not well understood [19,20]. There is no universal definition of community participation, though there are various forms, levels and interpretations of participatory approaches in PHC and water governance [21]. In general terms, community participation refers to involvement or engagement of people affected, or those who can affect decisions [22]. Community participation in both PHC and water governance, as mandated in formal policy and legislative frameworks, is of great importance for the realisation of universal health coverage (UHC), and the National Health Insurance (NHI), and national water security, and calls for an urgent reassessment of participatory practices in South Africa. Understanding the form, extent, context and dynamics of community participation and implementation is a critical gap in the evidence base in South Africa.

This paper sought to review evidence on community participation in PHC and water governance in South Africa. The main aim was to provide a narrative account of community participation in both sectors, based on existing literature, to help understand the realities and dynamics, and promote cross-sectoral learning to improve implementation. This paper explores why the concept of community participation, reflected so well in policies, is difficult to realize in practice, and what needs to be done to strengthen and support it. In literature, health and water are addressed separately, but it is important to consider both together for cross-sectoral learning.

## METHODS

### Search strategy

A narrative review was conducted to understand and describe how community participation through health committees, CMAs, WUAs, IBs and CMFs was implemented. Furthermore, an in-depth interpretation and critical reflection on the forms, extents, contexts and dynamics of participation were performed to understand how participants were involved in planning and decision-making responsibilities through such governance structures as health committees CMAs, WUAs, IBs and CMFs [23]. Governance refers to the processes applied to careful management of the wellbeing of population in a given system*** [24,25].

We did not follow a structured protocol for search, but we searched for articles reporting on community participation. In addition, there was no assessment of quality of individual studies. Peer-reviewed literature was found by searching databases using key search terms: community participation, community involvement, community activism, community engagement, PHC, health committees, water, CMAs, WUAs, IBs and CMFs. Other keywords were used based on synonyms and variations of terms of our topic (Supplementary materials 1). The databases included were Medline (Ovid), EMBASE, Google Scholar, Web of Science, WHO Global Health Library, Global Health and Science Citation Index.

A grey literature search was also conducted by searching libraries and websites of key water management and PHC-related institutions for additional material. Grey literature was important in this review to contribute to data not found in scientific/academic literature, fostering a more balanced picture of the evidence, while reducing publication bias [26–28]. These included grey literature from; the Department of Water and Sanitation, Department of Agriculture, Rural Development, Land, and Environmental Affairs, Department of Health, Health Systems Trust, World Health Organization (WHO), United Nations Children’s Fund (UNICEF), The Water Project, Life Water International, and International Water Resources Association. Additional relevant material was obtained by hand-searching and screening bibliographies of included studies, searching for key authors and experts in the field in South Africa.

Search terms were applied in different combinations depending on the source, using Boolean operators ‘AND’, ‘OR’ and ‘NOT’. Titles and abstracts were screened for relevance followed by full-text screening for relevant content. Both qualitative and quantitative studies were included. Mendeley reference management software was used to manage and store the literature.

### Eligibility criteria

All studies that involved the community, households, service users, public and their representatives in the planning, implementation, and monitoring of PHC and water services, or interventions in South Africa from January 1994 to June 2020 were included, dates coinciding with the post-apartheid era. In the domain of PHC, these included studies that involved the community in disease prevention, health promotion, healthy living, and/or health service delivery.

In participatory approaches to water governance, these included studies that involved community in water supply decisions, sanitation programmes, irrigation services and flood risk measures. Studies were excluded that involved individuals making decisions regarding personal service delivery outside health committees, CMAs, WUAs, IBs, CMFs, governance structures in PHC or water.

Language was restricted to English, and no restrictions were based on the study design. Due to heterogeneity of studies, in terms of study population, locations, participants and variations in measures of participation, results were thematically analysed. A narrative synthesis was then generated by developing a preliminary thematic synthesis of findings of included studies according to the analytical framework, described below.

### Analytical framework

Three conceptual frameworks of community participation were synthesised to identify and map existing literature on how participants were involved in the process of policy-making, decision-making and implementation in PHC and water governance in South Africa (Table 1) [22,24,29].

**Table 1.** Analytical framework: combined frameworks of community participation

Framework	Description and limitations	Framework Constructs	Constructs
Arnstein, 1969: Ladder of citizen participation	The framework, one of the best known, comprises eight rungs on a ladder, which relate to the forms and extents to which citizens are involved and have obtained decision making power. The bottom of the ladder, referred to as non-participation, include two rungs: manipulation and therapy. The third, fourth and fifth rungs which are informing, consultation and placation are described as tokenism. Under this category, participation may fail to affect outcomes and the status quo may remain. At the top of the ladder are three rungs, partnerships, delegated power, and citizen control, collectively referred to as degree of citizen power. At this level, participation is meaningful, and citizens have power in decision making and can affect change.	Manipulation, Therapy, Informing, Consultation, Placation, Partnership, Delegated power, Citizen control	Forms and extents
Cornwall, 2008: Participation meaning and practices	Cornwall argues that all forms and meanings of participation could be found in a single project or process at different stages depending on the context and dynamics of participation. Cornwall proposed that the following influence both participation and the outcomes: the intentions of those who initiated participation, claimed spaces, or invited spaces, who participates, who is excluded or who excluded themselves, influence, what activities people participate in, and at which stage in the process.	Context, Dynamics, created spaces, Invited spaces	Contexts and dynamics
Rifkin,1986: Lessons of community participation	Rifkin reviewed more than 200 participatory health programmes and developed a planning framework to improve community participation with four lessons. Firstly, it is practically impossible to have a universally acceptable definition of community participation given the complexity and dynamics of the process. Secondly, sustainable community participation processes cannot be established through health programmes alone, but require an integrated approach open to community priorities that may not be related to health. Thirdly, the political environment influences community participation. The fourth lesson is that it is not realistic to have a universal model for community participation programmes considering its context dependency. These lessons assisted in the development of three questions which are: Why participation? Who participates? And how do they participate? When answered, the questions would help address the dynamic process of community participation and assist clarifying and implementing programme objectives	Context, Dynamics	Contexts, Dynamics
Limitations of the frameworks	First, there are always power relations and inequalities at play. Those with authority in many cases find it hard to let go of power and recognize the voice of the marginalised. However, increasing participation is a crucial way in which capacity and authority can be acquired. The other limitation is that not everyone is willing to participate, and participation requires dedicated citizens. One cannot assume that participation might have a single outcome, unexpected delays might arise trying to reconcile conflicts and consensus processes might not adequately respect differences. Meaningful participation relies on adequate resources and quality information.		

Firstly, Arnstein’s (1969) classic framework of citizen participation is fundamentally based on the involvement of citizens and their power to make decisions [28]. According to this framework, participation can range from meaningless or marginal participation, to empowerment, where citizens develop power to contribute to solving challenges affecting their communities [28]. Secondly, Cornwall (2008) extends Arnstein to go beyond simplistic dichotomisation of community participation as applications that can be described as ‘good’ (empowerment) or “bad’ (tokenism) and encourages consideration of the contexts and dynamics of participation [29].

Finally, Rifkin (1986) derived four lessons from review of participatory health programmes, and developed a framework to respond to the dynamic process of community participation [21]. In doing so, she focused on three questions to consider when planning community participation programmes which are: Why participation? Who participates? And how do people participate [21]? We used Arnstein’s concepts of ‘forms and extents and Cornwall and Rifkin’s concepts of ‘contexts and dynamics’ as the lens for our review (Table 1). Underpinned by these analytical categories, the thematic analysis developed a grounded understanding of how participation has been implemented in practice. The contents of included articles were then compared.

### Data extraction and synthesis

Data extraction was guided by how communities participated in decisions about programs, and activities in the sectors of health and water since 1994 in South Africa. A systematic descriptive summary of included studies was performed by tabulating details about the study design, characteristics, context, process, and outcomes in Microsoft Excel.

Only literature reporting on health committees (also known as clinic committees and health facility committees) in PHC, and CMAs, WUAs, IBs and CMFs in water governance as formal structures for community participation were included for analysis. Only health committees were included in the health sector as they are relatively universal across all nine provinces in South Africa and are formal structures at the community level. Four different structures CMAs, WUAs, IBs, CMFs were included since they are intricately linked, and all include community-level stakeholders. These governance structures were spaces that facilitated communities to provide input, insight and feedback in the planning and organisation of services.

The articles and documents were read in detail. During extraction, the review team met biweekly to discuss the content of articles and themes emerging from both sectors and resolve inconsistencies in data extraction or interpretation of the studies. After extraction, data were collated and synthesised into themes derived from the analytical framework. A narrative synthesis was then developed to summarize the findings of different studies in relation to design, settings, contexts, processes, and outcomes reported. A preliminary synthesis was performed, translating data into themes. The results were discussed taking note of emerging patterns, then grouped into emerging themes on forms and extent, and contexts and dynamics of community participation in both sectors.

## RESULTS

**Figure 1.**
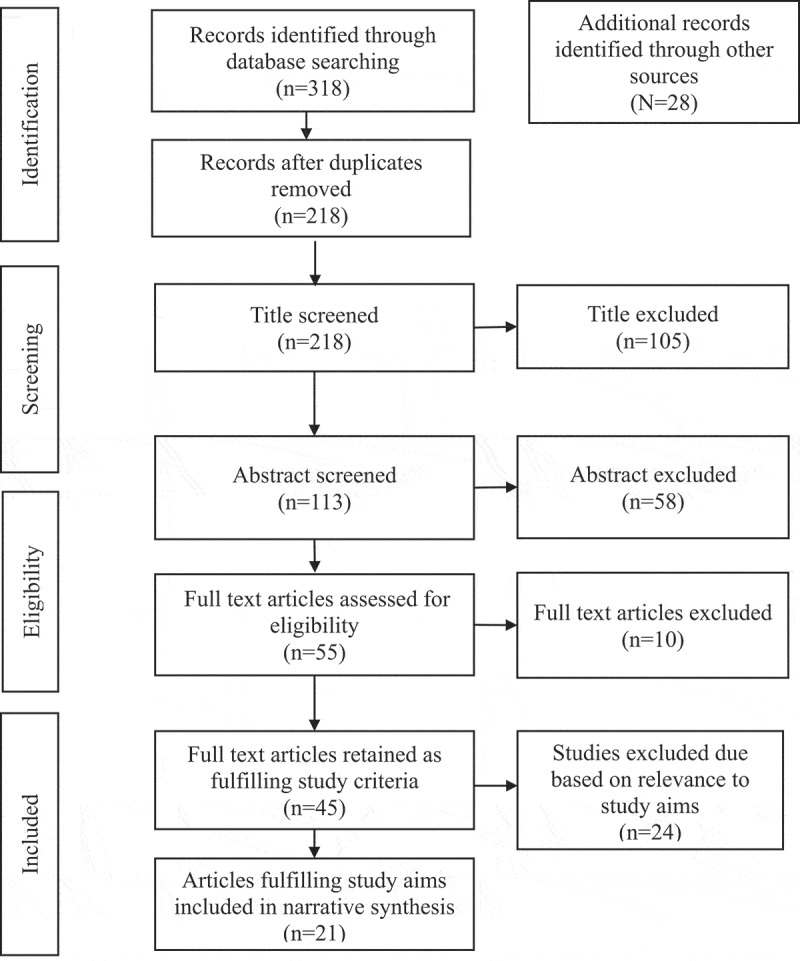
Literature search flow chart

In this section, we review how communities participated in these structures: health committees, CMAs, WUAs, IBs, CMFs in health and water sectors. Figure 1 provides a summary of the search process, including articles and documents. A total of 344 articles were identified, of which 218 remained once duplicates were removed (Figure 1). These were first screened by title, and the 113 left were then screened by abstract to ensure that they address community participation in PHC and water governance structures. The articles were further reduced to 45 after applying inclusion criteria. After screening for relevance to study aims and objectives, 21 articles met the eligibility criteria (Figure 1).

Of these, 11 discussed health committees as formal structures for community participation in PHC (Table 2). Generally, there was a dearth of information on health committees. Out of these eleven, six articles/documents focused on the existence and roles of health committees, while the other three discussed training, and two focused on challenges in developing health policy and value of community participation through health committees.

**Table 2.** Community participation through the establishment of health committees in Primary Health Care facilities across South Africa

Discussed by	Province	Study Design	Sample	Description
Padarath and Friedman, 2008	KwaZulu-Natal, Eastern Cape, Free State, Gauteng, Limpopo, Mpumalanga, North West, Northern Cape, Western Cape	Mixed methods: Cross-sectional survey Focus group discussions(FGDs)	Interviews conducted with facility managers in all nine provinces. 3 Focus group discussions with clinic committee members	The study investigates the existence and functions of health committees. Data was collected on the number of health (clinic) committees associated with public primary health facilities, composition, membership, and scope of activities. Data for 2003 and 2008 was compared to determine progress in health committees.
Meier et al, 2012	Western Cape	Qualitative: Policy analysis	Semi-structured interviews with key informants and stakeholder discussions	The study investigates the challenges in developing health policy for health committees in Western Cape. Documentary policy analysis and semi-structured interviews on the evolution of South African community participation policy.
Boulle et al, 2013	Western Cape	Mixed Methods Survey Qualitative	A survey held with 94 health committee members. Focus group discussions with clinic committee members were conducted with 14 clinics.	The study was conducted in Nelson Mandela district across 49 PHC facilities to understand the role of health committees and the challenges they face.
Haricharan, et al 2021	Western Cape	Qualitative	Interviews with key stakeholders, FGD with health committees	The study investigates the distribution and allocation of health committees, understand their functioning, and factors that influence functioning, training needs and recommendations to strengthen them. Health committees were identified through information from Cape Metro health forum eight subdistricts’ fora and speaking with PHC facility managers at individual clinics. Interviews were also done with key stakeholders and focus group discussions with three health committees.
McKenzie et al, 2017	South Africa	Case study	N/A	A case study in South Africa of Primary Health Care System Profiles and Performance (PRIMASYS) that aims to advance the science of PHC to support efforts to strengthen PHC systems and improve implementation, effectiveness, and efficiency of PHC interventions.
Haricharan et al 2014	Western Cape	Mixed Method: Survey Qualitative	Participant observation. In-depth interviews with key-stakeholders, FGDs and survey conducted with clinic committee members	The work was done to better understand the roles and functions of health committees in a re-engineered PHC system and the best institutional and the legal framework to maximise the contribution of health committees to a responsive health care.
Cleary et al, 2015	Western Cape	Qualitative	A series of reflective multi- stakeholders’ workshops including clinic committee members	The work was done at Mitchells Plain sub-district health system. A set of engagements were conducted to bring multiple stakeholders into conversation with each other. Community profiling and local action groups (LAGs) were developed for continuous engagement to strengthen the district health system through community participation. LAGs comprised mainly health committee members.
Mulumba et al, 2018	Eastern Cape and Western Cape	Intervention study	405 and 202 committee members in Western Cape and Eastern Cape respectively	Training intervention to enhance the potential of health committees. A training guide and an instructor manual was developed, and training was conducted with health committee members. Specific training activities included capacity building for health committee members, engaging with health officials and policy makers, building civil society networks, producing, and distributing educational materials.
Zwama et al, 2019	Western Cape	Qualitative	34 Health care providers from City of Cape Town health sub-districts	Evaluated a right-based, interactive training of health providers with health committee on relationship building, and governance, health provider authority and influence as well as how power imbalances affect health committee functioning. Health provider training aimed to establish and strengthen health provider’s relationships with health committees.
Esau et al, 2020	Western Cape	Qualitative	11 Managers and health facility supervisors; 7 participated in 2 FGDs and 4 in key informant interviews.	Explored the experiences of the training of the facilitator (ToF) learning programme in one district whether training was done according to the intention of ToF learning programme and whether selected trainers understood and were able to apply the training to the health committee.
Haricharan, 2015	South Africa	Qualitative	Telephonic interviews conducted with representatives of all nine provincial health departments	Reported the status of provincial health committees’ policies, draft policies or guidelines and support available to health committees.

**Figure 2.**
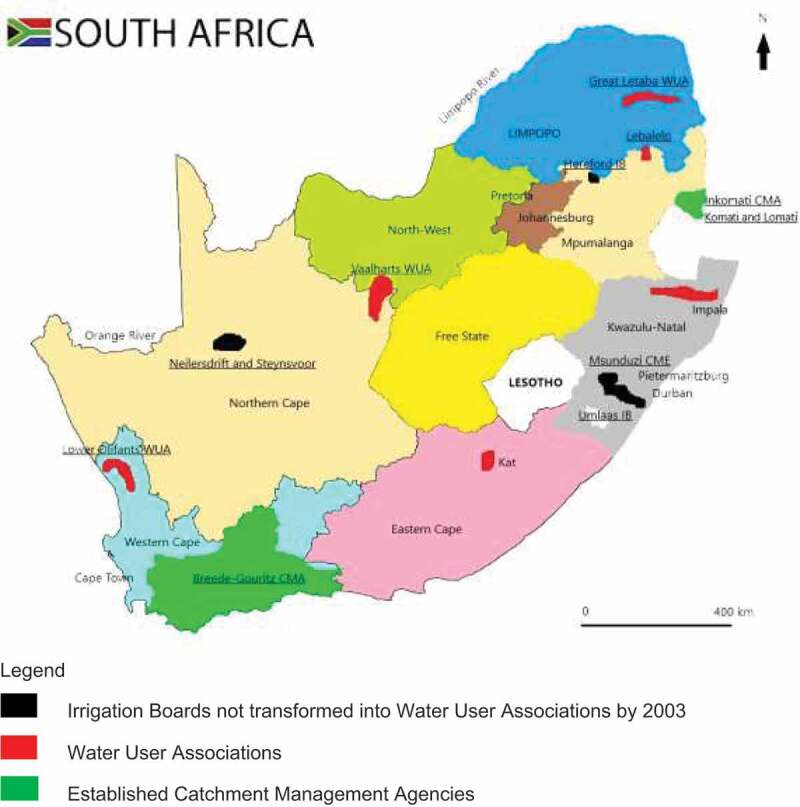
Map showing the location of water governance bodies included in this study

Source: Merged impression of water governance bodies as presented by Faysse, 2004 and Meissner, 2016.

In water governance, the 10 articles reviewed provided 14 case studies describing practices of participatory water governance of eight water governance bodies, comprising CMAs or WUAs/IBs in specified locations (mapped in Table 3 and Figure 2). The eight water governance bodies established in South Africa were Inkomati-Usuthu CMA, Breede-Gourizt CMA, Msunduzi River CMF, Lower Olifants WUA, Great Letaba WUA, Vaalhart WUA, Umlaas WUA and Hereford IB. The narrative review is presented according to key constructs from the analytical framework: forms, extents, contexts, and dynamics of participation.

**Table 3.** Participatory water governance bodies: Catchment Management Agency (CMA); Water User Association (WUA); Irrigation Board (IB) and Catchment Management Forum (CMF) included in this review

Discussed by	IB/WUA/ICMA	Province	Description
Boakye et al, 2012	Msunduzi CMF	KwaZulu-Natal	Exploring the involvement of previously disadvantaged and marginalised communities in Catchment Management Forums (CMF).
Boakye et al, 2012			Investigating the extent of participation of previously disadvantaged in water management.
Chibwe et al, 2012	Inkomati -Usuthu CMA	Mpumalanga	Understanding water reform process and factors behind outcome of decentralization process of Inkomati Usuthu CMA.
Brown, 2012			Exploring the potential of participation to change geography of water.
Brown, 2011			Exploring the institutionalization of participatory water resource management in post post-apartheid South Africa.
Denby et al, 2016			Examining how the efforts at implementing Integrated Water Resource Management translate into practice and the interpretations, challenges and outcomes surrounding the implementation are understood and affect people.
Brown, 2014			Assessing what can and cannot be expected from participation through comparisons and differences between processes and outcomes.
Seshoka et al, 2004	Lower Olifants WUA	Western Cape	Lower Olifants WUA and historically disadvantaged individual needs, degree of decentralization, transformation process and Integrated Water Resource Management practices.
Seshoka et al, 2004	Great Letaba WUA	Limpopo	Establishment of Letaba WUA, water management issues, water users, waterworks, and management practices of the Letaba WUA.
Seshoka et al, 2004	Vaalhart WUA	Northern Cape and North west	Transformation and degree of involvement of historically disadvantaged individuals.
Fayse et al, 2004	Umlaas WUA	KwaZulu-Natal	The extent to which the need for historically disadvantaged individuals could be satisfied by the WUA. Establishment of the Irrigation Board into a WUA and current and future involvement of historically disadvantaged individuals.
Fayse et al, 2004	Hereford IB	Mpumalanga	Management of Hereford IB and involvement of the historically disadvantaged individuals in the Hereford IB.
Meissner et al, 2016	Breede Gourizt CMA	Western Cape	The establishment of Breede-Overberg now known as the Breede-Gourizt CMA, the politics and strategies involved in its establishment.

*Forms and extents of community participation*: The form and extent of community participation in health and water governance explored the degree and level of involvement in identifying and defining problems facing their communities, identifying solutions to address the problems, and implementation of programs. In this review, the forms and extent of community participation were described in terms of coverage, roles and responsibilities, skills, and capacities.

### Coverage

A total of seven (64%) out eleven of the retrieved studies on health were conducted in the Western Cape [9,30–35], though the Eastern Cape was the first to have a policy in place for health committees in 1999 [36]. The Western Cape draft policy to establish health committees was developed in 2008, but there were implementation challenges, and not all PHC facilities in the province had a health committee by 2014 [7,30]. With regards to coverage, in 2008, 60% of PHC facilities in the country reported having a health committee [37]. Free State Province reported having the highest coverage, at 78%, followed by the Eastern Cape with 73%, while the lowest percentage, 31%, of facilities having health committees, was reported in Mpumalanga [37]. More recently in 2019, the situation seems to have improved with 100% of clinics having health committee in 5 provinces (Table 4) [38].

**Table 4.** Number of health committees per province, and their responsibilities

	Province								
	Eastern Cape	Free state	Gauteng	KwaZulu Natal	Limpopo	Mpumalanga	Northern Cape	North West	Western Cape
Number of Health Committees	696	139	1488	592	492	263	169	301	200
% of clinics with health committees	95	46.3	100	100	100	83	100	100	46.7
Average members per committee	15	7	8	15	9	12	5	7	12
General roles and function of health committees	Oversee adherence and provision of PHC packages, identify health problems in community, monitor and report extent to which the PHC facility meets health indicators and targets, performance management, monitor how the PHC facility manage complaints submitted by patients and communities, hold management accountable for implementing decisions made during committee meetings, foster partnerships with other community stakeholders, fundraising for PHC activities, raise and manage Health committees funds, advocate for use of PHC facilities as the first point of entry of health care, provide a platform for the patients and community grievances, social mobilisation, be visible, build relationships with PHC staff.								
Composition	Community members, community representatives (for example representatives of certain sectors such as traditional health council, NGOs, women’s group etc.), facility manager or clinic staff and local ward councillor. The national average for health committees is 10 members.								
Appointment	Election of community representatives vary across provinces, some are elected during community meetings, or volunteering, others are elected by the local councillors, or health committee chairperson or clinic staff.								
Finances	Health committees not much involved in planning and budgeting and expenditure processes, though they are required to raise funds to support their activities. They are expected to execute certain activities with no budget provided.								

In 1999, the country was divided into 19 Water Management Areas according to river catchment systems, not administrative boundaries, referred to as Catchment Management Agencies (CMA). These 19 CMAs were reduced to 9 in 2012 for logistical reasons [19,39]. Besides CMAs, community-based structures were formed such as Water User Associations (WUAs) and Catchment Management Forums (CMFs) [20,21]. Some pre-apartheid Irrigation Boards (IBs) were transformed into WUAs. Decentralization of water resource management to catchment level was to ensure incorporation and participation of all stakeholders (farming, non-farming, water licensed and non-licensed water users alike), especially historically disadvantaged individuals.^1^^1^A South African citizen due to apartheid policy that had in place, had no voting rights in national elections prior to the introduction of the constitution of the Republic of South Africa, 1993 (Act No. 200 of 1993).

### Roles and responsibilities

8/11 (73%) studies from 2008 to 2020 indicated that health committees lacked understanding of roles and function [31–34,37,40,41]. In provinces, according to policy frameworks, health committees were established to promote community participation through their roles and functions of governance, oversight, advocacy, collaboration, social mobilization and representation of community needs [42]. In all nine provinces in South Africa, studies conducted in different years from 2008 to 2019, found that some established health committees were not fully functional and committee member turnover was high [32,38,40,43]. Committee members often left for better opportunities or relocated to other provinces, due to lack of stipend [31,32,42,44].

Even though the Eastern Cape had the most detailed draft policy on roles and functions of a health committee, the literature indicated challenges with implementation [36]. Reviews of health committees in Nelson Mandela Bay health district and in the greater Cape Town Metropole in 2013, found that health committee members were unaware of the policy (Western Cape Draft Policy) hence there was a gap between policy and practice [32,45]. In addition, PHC facility managers did not understand their own roles, as most of them did not produce mandated reports to the committees [32].

In all nine provinces, health committee services were reported to exist on the periphery of the health system, hence not effectively fulfilling their mandate to enhance community participation. The health committees in all studies reviewed could be classified by Arnstein’s ladder as having a variety of ‘degrees of tokenism’ [9,30,32–35,37,40,41,44,46]. In all nine provinces, health committees had limited participation regarding contributions towards clinic planning and delivery of health services [7,30,38,47]. Health committees as formal structures for community participation lacked authority to function properly, for example in addressing health issues in the community or monitoring service delivery and quality, as they were not fully integrated into the health system [36,48].

In particular, reported evidence in the greater Cape Town Metropole indicated that health committees had limited participation as they were not involved in decision-making processes, setting the agenda, identifying problems, or finding solutions [44]. Their roles were mainly supportive and focussed on providing service to the clinics, e.g. assisting clinics in day-to-day running, projects and health awareness [44]. It appeared that not only health committees lacked clarity of their roles and responsibilities, but service providers were also not aware of the mandate of the health committees [44]. Lack of clarity on roles and responsibilities caused health committees to feel inadequate and members reported that they consider their role in decision-making limited regarding service delivery in PHC facilities [31,32].

Five case studies on the Inkomati-Usuthu CMA were from 10 articles covering the period from 1994 to 2016. Participatory processes began in 1997 in Mpumalanga province, with the NWA providing a mandatory basis to engage all stakeholders to work together to ensure equitable access to, and distribution of, water basin resources [47]. The findings showed that participatory water governance is limited nationally. The forms and extents of community participation were reported to be based on inclusion and engagement of historically disadvantaged individuals in planning, comprehension and articulation capacity of stakeholders and decision-making power. Despite this, in all 14 case studies reviewed, the role and involvement of various stakeholders was not a straightforward process, and it was not always feasible to include all stakeholders in the establishment of CMAs [18,47,49–56]. The difficulty was not only in managing conflicts of interests, power imbalances and time from various stakeholders but also the administrative issues such as finances, human resources, language used, and venues of meetings [18,47,50,51,53–56]. Participation in water governance was also tokenistic according to Arnstein’s ladder of participation.

### Skills and Capacity

One study in this review indicated that more than 50% of health committees were not functioning in Nelson Mandela Bay Municipality in 2006 [32]. The health committees were not producing reports as they should have since they lacked understanding of health targets and indicators and monitoring of the PHC package was seen as an insurmountable task. Limited skills and capacity inhibited effective functioning as committees could not perform governance functions, although they were active in advocacy, bringing health-related problems from local communities to the attention of PHC facilities [36,37], and social mobilisation [9,32,42].

In seven (64%) out the 11 studies conducted in different years from 2008–2019, health committees were reported active in support of calendar health days, mobilising communities to participate [36,37]. Calendar health days are international, regional, and local health awareness events, e.g. World AIDS day and anti-tobacco week. Fundraising was a key challenge, as most did not know how to fundraise, even though it was their role, and they lacked clarity on whether they should raise funds for their own functions or to support PHC services [38].

Regarding being accountable to the community and local organisations, the feedback process was reported to be informal as they lacked clarity on how it should be conducted. The Western Cape lagged behind in implementing the legislation [45]. Even though health committees were supposed to provide a link between the community and the health system, eight studies reviewed indicated that their influence in decision-making, prioritization and implementing health services specific to their community were limited [31,32,34,40,41].

In Lower Olifants WUA, lack of knowledge and experience in commercial farming practices among historically disadvantaged individuals was planned to be mitigated by capability building. However, by 2003 training and support to the majority of historically disadvantaged individuals was not enacted as per the WUA business plan [53]. Additionally, historically disadvantaged individuals lacked capacity and competence in dealing with water management affairs, compounded by the inability to understand the concepts and technicalities of the NWA, CMA reports and other materials [18]. Further, a majority of water users, especially historically disadvantaged individual small scale and emerging farmers, did not receive the amount of water that they were entitled to and had paid for, resulting in mistrust between the Letaba WUA and stakeholders [50].

*Contexts and dynamics of community participation*: This explored the contextual factors’ influence on participation and how this in turn affected success or failure of programmes or interventions. Context and dynamics of participation are presented under the following sub-themes: representation, institutional support, quality of information, and access to information and accountability.

### Representation

In the Eastern Cape, representation affected the way a health committee should function. Members were mainly composed of volunteers who were entitled to a monthly stipend. In seven (64%) out of the 11 articles conducted from 2008–2019, it was reported that stipends were not forthcoming, and health committee members became tired of volunteering. Interestingly, few health committees had local government councillors and PHC facility staff as members, as required by legislation [30]. Within the PHC facilities, health committee members highlighted the need to be formally recognised, and requested name tags [32].

Literature on the Inkomati-Usuthu and Breede-Gourizt CMAs indicated that enactment of the NWA created space for early stakeholder engagement, in 1997 but that mainly white farmers took an active role in establishing the CMAs and transforming the WUAs [18], severely limiting space for diverse participation [48]. In the Letaba WUA, participatory water governance was characterised by the exclusion and eventual misrepresentation of stakeholders such as farm workers and non-farming industries in the management committee meetings, despite their expressed desire to participate [50].

Gender exclusion was another key issue identified affecting participation in historically disadvantaged individuals, especially women [50]. Management committees were 60% male dominated and 90% of stakeholders reported that gender issues were not a consideration in management committee meetings [53]. In addition, stakeholders in Letaba WUA such as municipalities and representatives of game reserves stopped attending management committee meetings due to deliberation lacking relevance to their line of work, while others, such as worker’s unions, were not even considered in the initial public participation process [50]. Reluctance of some key stakeholders to participate in the activities and operations was a barrier to participation. The Nkomazi local municipal authority was unwilling to participate because joining the WUA would raise the cost of accessing water [18].

The management committee in Lower Olifants WUA was reported to have good representation of all stakeholders, including historically disadvantaged individuals and small-scale farmers [50]. The Vaalhart WUA created a constitution that ensured all members had equal voting power of one vote per member, and that gender representation was addressed. This resulted in an increase in women representatives on the management committee hence, this WUA was highly considerate towards gender issues regarding meeting locations, schedules and times compared to other WUAs [50].

The venues for Inkomati-Usuthu CMA meetings were also potentially inappropriate and included ‘high end’ hotels and resorts far from former rural homelands and in most cases no consideration of the interests of historically disadvantaged individuals, cost of attending meetings and potential loss of earnings [49]. Conflicts of interest over representation were reported among stakeholders and this resulted in the Inkomati-Usuthu CMA and WUAs experiencing operational challenges six months after establishment [18,20,21,42,46]. Additionally, some relevant stakeholders did not continuously attend meetings due to fatigue after being asked to commit to the same process over and over without seeing change.

### Institutional support

Despite wide coverage, there were no studies available on how health committees’ function in provinces other than Eastern Cape, Western Cape and Gauteng. Nevertheless, these described limited participation due to lack of political commitment, lack of support from the health system, lack of resources and limited participation by facility managers and local government councillors [8,35]. Without support, health committees cannot do their jobs properly.

Located in the Western Cape, the Lower Olifants WUA was the first WUA to be successfully established in South Africa in 2000 [56]. An account of participatory water governance from Lower Olifants WUA shows the early participation of the Vredendal IB, which acted as a founding member of the Lower Olifants WUA during the IB transformation process. Accounts of participatory water governance from Msunduzi CMF disclosed the presence of conducive social spaces that facilitated social cohesion, learning by doing and opportunities for knowledge transfer among participants [54]. It was reported that participants commended the Department of Water and Sanitation on the public participatory techniques used to engage and disseminate information to stakeholders in the meetings. Each meeting had agenda items to which stakeholders were at liberty to add concerns or other items before meetings began. Additionally, stakeholders were free to interact with the chairperson [54].

### Quality of information and access to information

The literature on water governance revealed that, while a great effort was made to empower communities and historically disadvantaged individuals, no consideration was made to make the process sufficiently transparent to stimulate meaningful participation. For example, Some stakeholders in CMAs purposefully concealed information for their gain. [55,57,58]. In four WUAs and two CMAs, the language of communication was not an issue as most marginalized community representatives were able to communicate efficiently in Afrikaans, which was used as the bridging language, together with English [44,45,49].

Moreover, feedback was often poor, and stakeholders were dissatisfied with access to information before meetings [50,56]. Firstly, there was the lack of expertise and experience of the Department of Water and Sanitation staff to ensure that CMA establishment proposals were handled and reviewed within reasonable time-frames [48,49,54]. Secondly, lack of feedback to communities from CMA representatives was noted, resulting in the lack of awareness of key catchment water discussions and processes which perpetuated an environment of mistrust among representatives and communities [49].

### Accountability

Our review identified that though health committees are statutory structures, they had no power to enforce decisions arrived at in meetings. On the other hand, they lacked capacity in financial resources and administrative skills as a result they failed to meet their responsibilities [7,38]. Therefore, institutional inertia and capacity limitations affected the willingness of committee members to continue as they did not see the legitimacy of being involved. Lack of accountability and consideration to deal with these matters cripple meaningful participation in health committees.

In Breede-Gourizt CMA, the principles of respect, integrity reliability and accountability were supposed to influence decisions and actions of this CMA employees and board members [48]. In this view, this CMA was accountable to the Minister and stakeholders although the accountability was skewed towards the Minister [48]. The Minister received regular reports compared to other stakeholders [50,51]. In Msunduzi CMF, participants reported challenges in consensus decision making due to emerging farmers lacking skills, knowledge, resources and experience, hence they were dominated by commercial farmers [50,51]. Also in Lower Olifants WUA, community representatives reported that the voices of their communities were not being heard and included in the decision-making processes [50]. Upon further analysis of participatory water governance in Inkomati, several observations during and after the formation of the CMAs and WUAs were made. There was influence of partisan political leaders, who pressured the Department of Water Affairs and Forestry (now Department of Water and Sanitation) to ensure one CMA was established before the 2004 elections [59].

The absence of systems and mechanisms to ensure transparency and accountability of water management institutions and Community-Based Organisations was shown by the immediate disbandment of the Inkomati-Usuthu CMA Advisory Committee after recommendations had been drawn, resulting in a lack of accountability, as the board could not be held accountable [18,49]. In Vaalhhart WUA, limited participation and engagement of some stakeholders (farm workers) in basin processes, was reported to arise from the disconnect between farm workers and their representatives, due to lack of internal organisation of the farm-worker community [50].

## DISCUSSION

Community participation is assumed to contribute to a: ‘process of democratization and empowerment’ [60]. This review highlights, during the early years (from 1994), significant policy recognition and support for community participation. Constitutional, legislative mandates and policy directives supported institutionalization of community participation in both water and health. This was an important process in South Africa to redress structural inequalities linking service delivery to communities. Despite this, however, as policies became established, inter-related structural and implementation barriers to effective and meaningful participation were seen.

In water governance, from 1996–2016, mandated spaces were autonomous, well-funded and better structured than in the health sector. However, progress with implementation was undermined in later years, owing to problems with representation, power imbalances, and low capacity and skills. These resulted in participation being mostly tokenistic, and the ability to influence change and empower proved minimal.

During implementation from 2008–19, health committees had *both* structural and implementation challenges including lack of power and authority to influence decision-making, lack of recognition by the health system, and lack of skills and capacity. Consequently, health committees ultimately departed from the original mandate to represent and advocate for community priorities and needs.

Formalised governance spaces have the potential to promote strong legitimacy, better-informed stakeholders, confident, committed, and skilled staff, and stakeholders [61]. Our review identified significant policy/implementation gaps, demonstrating that community participation is not just about creating structures but functionally and sustainably transferring power and authority to marginalized people. These findings reflect the need for sustained processes focussing on improving authority, representation, and resources for effective and meaningful participation.

The results are consistent with other work from South Africa on challenges of sustainability and functionality of health committees [62,63]. These issues are also not limited to South Africa, existing internationally, with participation via health committees described as mostly tokenistic, departing significantly from original mandates in all the provinces [19,64–66]. Moreover, to date only two provincial CMAs (of a total nine) have been established in South Africa. Stuart-Hill *et al.* observed that this is due to the complexity of engaging diverse stakeholders, even though highly inclusive and learning-orientated [67]. Good mechanisms for community participation are hard to establish and co-production takes time [63,64].

Although formal spaces were established more systematically in the water sector, they were often open to ‘elite capture’, lobbying and tension among diverse stakeholders. The findings demonstrate that inclusion and representation do not always equal participation or engagement. They can present opportunities for more powerful stakeholders to take advantage and mould structures and processes of deliberations to serve their own interests, consolidating positions of power and control over those with less autonomy, agency, and representation.

The findings also indicated that participatory processes are not always transparent. In this scenario, participation may do more harm than good when they are ‘equivocal’ and ‘disorderly’ [68,69]. Popay et al. (2020) argued that for community participation to achieve its potential and reduce inequities, there is need to support disadvantaged communities through capacity building to exercise greater control over decisions and actions [70]. This can be achieved when the ‘empowerment process actively engage[s] with power dynamics operating in community settings’ for sustainability’ [70].

The findings demonstrated that health committees established from 2008–19 were, in many instances, involved to a limited degree in planning and decision-making due to structural and implementation barriers. Health committees lacked substantial voice within the health system. Lack of clarity on roles and responsibility has been a challenge in other countries [62,71–73]. The literature reflected the importance of community empowerment through capacity building and access to information [64,70,74,75].

These results support findings by Meier *et al.* (2012), who identified that communities are often required to participate in highly structured activities, without sufficient knowledge of the health systems, nor the skills to challenge powerful stakeholders and institutional processes on board and make their voice heard [9]. Under such circumstances, participants cannot take advantage of opportunities to impact decision-making, central to the success of community participation programmes [30,70,71].

Morrison and Dearden (2013) caution that community participation can be ‘compromised by the very contexts in which are meant to empower’ such as who initiates participation, who benefits, who participates, what influences they have, and which activities and at which stage in the process they participate [24,70,75]. Structures to support community participation often achieve less in practice than intended [70,76,77].

In our review, stakeholders in water governance were involved early in planning phase to co-produce, design and implement water delivery services. In PHC, health committees were involved in decision-making at the implementation stage, where they were involved in supportive roles with limited influence on service delivery and monitoring. Nevertheless, as described above, there remained a clear gap between policy and practice with little guidance on how to effectively implement sustain and expand participation over time. A literature review on stakeholder participation in environmental governance found that effective participation is hindered by involving communities in decision-making late, during implementation and not in the earlier identification and preparation phases [78].

Another review by Haldane *et al.* (2019), highlighted that exploring participation *as a process* is key, and could influence better health outcomes while dealing with issues of power or control, representation, resources and sustainability [10]. Early engagement and incentives may also help in promoting participation to ensure ownership and inclusive representation [78,79]. In Mexico, for example, farmers were actively involved in decision making for water governance because the majority were generating wealth from commercial farming [79].

The findings demonstrate limited insights into best practices for implementation, indicating how high degrees of citizen power were difficult to achieve and sustain. Creation of more conducive ‘invited’ spaces (i.e. within formal governance and planning) with careful consideration of the needs of stakeholders, and empowerment, would facilitate key relationships, processes, learning, networking and appropriate use of power to sustain participation. Thompson *et al.* (2012) substantiates the need for professionalization of community participants through capacity building to increase ability and power to meaningfully participate [80]. Capacity building has the potential to increase the control the communities could have over decision-making to become equal partners [70].

Finally, globally, the COVID-19 pandemic highlights the need for community participation to ensure realistic and appropriate responses to the needs of every community [81,82,83]. Community participation has not yet been well utilised in South Africa, however, with discrepancies between policy and reality compromising the roles of health committees as platforms for community participation, and linkages between communities and health system [9].

## Limitations

The literature was not exhaustive and was limited to community participation in health committees, CMA, WUAs, IBs and CMFs, health and water governance structures and gives a broad overview of community participation. We did not follow a predefined protocol for searching articles. Articles reporting on community participation outside these structures were excluded. Grey literature/unpublished reports were included in this review but an exhaustive grey literature review on the internet is probably not possible. Therefore, it is likely that some articles could have been missed. In addition, there were few articles reporting on community participation in health communities’ governance structures in some provinces. Many of the studies identified were from the Western Cape. In addition, there were few current studies reporting on the status of health committees in the country. The strength of our review is that it draws on multiple data sources and the first review on community participation in both health and water governance to be conducted in South Africa.

## CONCLUSIONS

The review demonstrates multiple threats to community participation. Even with clear policy mandates, risks remain. In both health and water governance, the establishment of participatory governance structures aimed to enhance participation and meet the needs of communities and previously disadvantaged individuals. Despite CMAs being better structured, better financed and engaging diverse stakeholders, they have not met the needs of communities and previously disadvantaged individuals in South Africa. On the other hand, health committees have limited participation, and focus on supporting clinics rather than representing community needs and priorities.

The study therefore recommends that those with power should ‘let go’ when they must. This includes recognising and valuing local knowledge. Community participation also needs power and authority of marginalized people for co-production of knowledge, ideas, and collective action and decision-making. Additionally, responsibility should come with capacity to be accountable. Hence issues of authority in decision-making, trust, resources, and capacity need to be addressed for effective participation and sustainability. The establishment of structures and mechanisms for enhanced participation should be seen as a gradual process which takes time and iteration to achieve its goals. Practical guidance is urgently required on how to put policy mandates into practice, sustaining and linking mandatory participation governance structures to wider governance systems to improve service delivery.

## Supplementary Material

Supplemental Material

## Paper context

In South Africa, health, and water policies support community participation. Community participation that is inclusive of marginalized groups and context specific is fundamental to equitable provision of health and water services. Implementation of community participation is deeply challenged in both water and health sectors in different but overlapping ways in South Africa. Institutionalization of community participation requires communities to be provided with adequate responsibility and authority while accountable to the results they produce to be effective.
