# Supplementary figures and images for: Lessons from community participation in primary health care and water resource governance in South Africa: a narrative review
Source: Glob Health Action. 2022 Jan 7;15(1):2004730. doi: 10.1080/16549716.2021.2004730 (PMC8745361; doi:10.1080/16549716.2021.2004730)

SUPPLEMENTARY MATERIAL/ Appendix 1

Supplementary Material :1


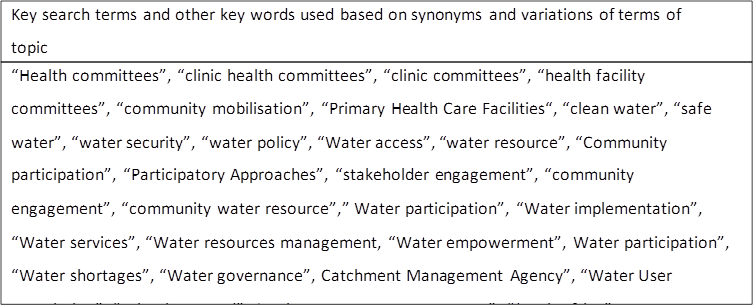

Supplement: Supplemental Material [file ZGHA_A_2004730_SM1697.docx]
